# A dual attention and multi-scale fusion network for diabetic retinopathy image analysis

**DOI:** 10.3389/fmed.2025.1614046

**Published:** 2025-09-08

**Authors:** Menglin Zhang, Qi Liu, Jialei Zhan, Jinwen Gao, Dong Xie, Jialang Liu

**Affiliations:** ^1^School of Nursing, Changchun University of Chinese Medicine, Jilin, China; ^2^Department of Applied Physics, Institute of Energy Research, Jiangxi Academy of Sciences, Nanchang, China; ^3^Laboratory for Big Data and Decision, National University of Defense Technology, Changsha, China; ^4^Faculty of Nursing, Wenzhou Medical University, Wenzhou, China

**Keywords:** medical image classification, multi-scale feature fusion, dual attention mechanism, adaptive feature representation, deep neural networks, lesion recognition

## Abstract

Robust classification of medical images is crucial for reliable automated diagnosis, yet remains challenging due to heterogeneous lesion appearances and imaging inconsistencies. We introduce DWAM-MSFINET (Dual Window Adaptation and Multi-Scale Feature Integration Network), a novel deep neural architecture designed to address these complexities through a dual-pathway integration of attention and resolution-aware representation learning. Specifically, the Multi-Scale Feature Integration (MSFI) module hierarchically aggregates semantic cues across spatial resolutions, enhancing the network’s capacity to identify both fine-grained and coarse pathological patterns. Complementarily, the Dual Weighted Attention Mechanism (DWAM) adaptively modulates feature responses in both spatial and channel dimensions, enabling selective focus on clinically salient structures. This unified framework synergizes localized sensitivity with global semantic coherence, effectively mitigating intra-class variability and improving diagnostic generalization. DWAM-MSFINET achieved 78.6% Top-1 accuracy on the standalone Messidor dataset, demonstrating robustness against domain shift. DWAM-MSFINET surpasses state-of-the-art CNN and Transformer-based models, achieving a Top-1 accuracy of 82.59%, outperforming ResNet50 (81.68%) and Swin Transformer (80.26%), while inference latency is 16.0 ms per image (not seconds) when processing batches of 16 images on NVIDIA RTX 3090, equivalent to 62.5 images per second. These results validate the efficacy of our approach for scalable, real-time medical image analysis in clinical workflows. We have released our code and datasets at: https://github.com/eleen7/data.

## 1 Introduction

**Figure 1:**
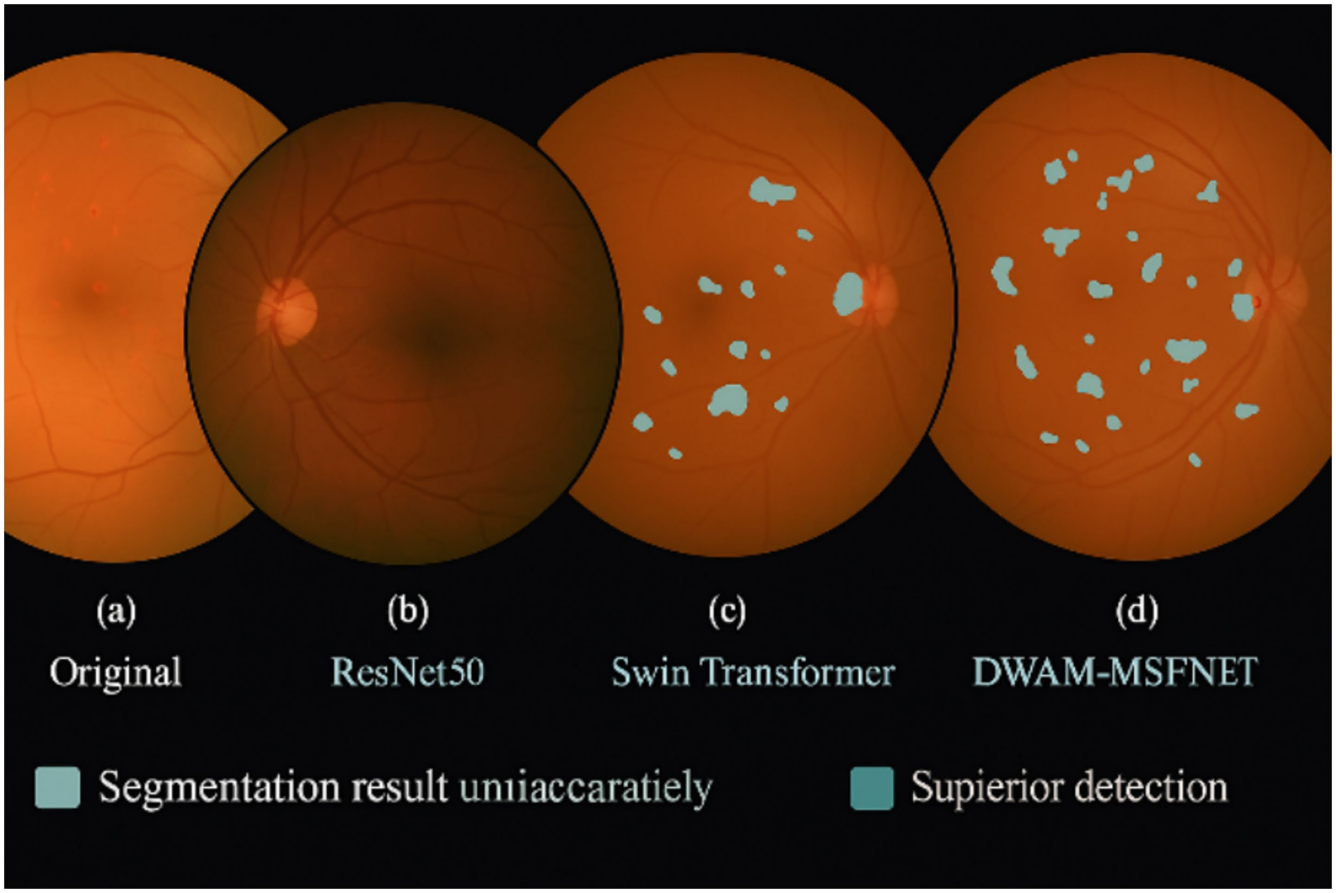
Visual comparison of diabetic retinopathy (DR) lesion detection across different backbone methods. **(a)** Original fundus image with mild DR symptoms. **(b)** Segmentation result using ResNet50, failing to highlight several subtle lesions. **(c)** Swin Transformer shows partial lesion enhancement but misses early-stage signs. **(d)** DWAM-MSFINET achieves more precise localization and clearly identifies small lesions, demonstrating its advantage in handling fine-grained retinal abnormalities. Key abbreviations: DWAM (Dynamic Window Adaptation Mechanism), MSFI (Multi-Scale Feature Integration), and DR (diabetic retinopathy).

Diabetic retinopathy (DR) is a serious vision-threatening complication of diabetes and a leading cause of preventable blindness worldwide (1). According to the World Health Organization, more than 460 million adults globally have diabetes, and the incidence of DR is expected to rise as diabetes cases increase (2). If left untreated, DR can progress to severe vision loss, underscoring the urgent need for early detection and timely intervention (3). Early diagnosis and treatment can halt disease progression and prevent complications such as diabetic macular edema (DME) and proliferative diabetic retinopathy (PDR), both of which can result in irreversible blindness (4). Recent advances in retinal imaging technologies, including high-resolution fundus photography and optical coherence tomography (OCT), have improved the screening and diagnosis of DR (5, 6). Despite these advancements, manual examination of the large volumes of retinal images remains challenging for clinicians due to the subtlety and variety of retinal lesions and the heavy workload (7). DR lesions vary widely in size, shape, and texture, making tasks like lesion segmentation and classification difficult and observer-dependent. In recent years, machine learning (ML) and deep learning (DL) techniques have gained traction for automated DR diagnosis (7–12). Traditionally, DR detection relied on manual assessment of retinal images by experts—a time-consuming process limited by the clinician’s experience and subjectivity. The advent of deep learning has introduced powerful new approaches for early DR detection and management (13). Machine learning algorithms can learn patterns from data to make predictions or decisions (14). For instance, Venuganth et al. explored ML techniques for DR diagnosis, highlighting the critical importance of early detection and timely treatment in preventing vision loss (15). Deep learning, a subset of ML using multi-layer neural networks, has shown superior performance in analyzing complex image data by automatically extracting hierarchical features. Convolutional neural networks (CNNs) in particular have achieved notable success in DR detection from fundus photographs. Prior works have employed architectures like VGG-19 and transfer learning to improve DR classification, especially when combined in ensemble frameworks (9, 16). A recent study in 2025 demonstrated the use of quantitative wide-field angiography combined with ML to assess DR severity, underscoring the potential of data-driven methods in quantifying retinal pathology (17). Another study evaluated AI-based DR screening in diverse populations, finding significant benefits of automated screening for early detection and management of DR (18). These efforts collectively indicate that DL models, especially CNN-based, can achieve high accuracy in identifying DR and even subtle retinal lesions (19). However, traditional CNN-based methods have inherent limitations. CNNs typically use fixed-size receptive fields and single-scale feature extraction, which can hinder the detection of small, early-stage lesions (20). While CNNs excel at learning localized features, they struggle to capture global context, potentially missing distributed or subtle patterns of disease across the retina. This lack of global awareness can result in incomplete lesion detection, especially in early DR when signs are faint and scattered. To address the limitations of fixed receptive fields, vision Transformer architectures have emerged as a promising alternative. Notably, the Swin Transformer introduced a shifting window-based self-attention mechanism that adaptively extends the receptive field and enables multi-scale feature representation (21). By partitioning the image into local windows and periodically shifting these windows, Swin Transformer can focus on the most relevant regions and also incorporate broader context, improving the detection of lesions at various scales (21). Compared to conventional CNNs, such transformer-based approaches better capture both local details and global structure, facilitating the detection of subtle microaneurysms and small hemorrhages while retaining awareness of the overall retinal image (22). The ability of Swin Transformer to adaptively adjust its attention window gives it a significant advantage in overcoming CNNs’ limitations in global information capture, especially in complex retinal images with lesions of different sizes and stages. In this study, we propose a new deep learning framework for DR classification that integrates contextual and multi-scale information capture within a unified architecture. Our approach specifically introduces two key innovations: the Dynamic Window Adaptation Mechanism (DWAM) and Multi-Scale Feature Integration (MSFI). DWAM dynamically adjusts the self-attention window size based on the local image context, enabling the model to focus on subtle lesion details in high-variance regions while still considering broad context in smoother regions. MSFI, on the other hand, employs multiple convolutional kernels of different sizes to extract and fuse features across multiple scales, effectively capturing lesions ranging from microaneurysms to large exudates. By combining these two mechanisms in a DWAM-MSFINET architecture, our method addresses the shortcomings of conventional approaches: it refines the receptive field on-the-fly for nuanced lesion patterns and concurrently processes heterogeneous lesion sizes. This synergistic design significantly improves DR lesion detection and classification, particularly for early-stage disease, thereby enhancing diagnostic accuracy and robustness. Moreover, the model is designed with efficiency in mind, paving the way for real-time, automated DR screening that can alleviate the clinical workload and reduce the global burden of diabetes-related blindness. As illustrated in Figure 1, existing CNN-based and ViT-based approaches often suffer from inadequate sensitivity when detecting small or early-stage retinal lesions. While ResNet50 misses micro-lesions entirely and Swin Transformer shows limited improvement, our proposed DWAM-MSFINET provides significantly enhanced visual localization, especially for subtle pathological features. This motivates our architecture design, which integrates multi-scale fusion (MSFI) and dynamic attention adaptation (DWAM) to improve lesion detection accuracy and robustness in complex fundus images.

The major contributions of this work are summarized as follows:

1. **Novel Transformer-Based Framework:** We propose DWAM-MSFINET, a new Transformer-based network for DR classification that achieves superior performance. The proposed model attains a Top-1 classification accuracy of 82.59%, exceeding that of baseline architectures (e.g., 80.26% with Swin Transformer), while also reducing average inference time to 16.0 s (versus 20.1 s for the baseline), making it suitable for real-time clinical deployment.
2. **Dynamic Window Adaptation Mechanism (DWAM):** We introduce DWAM to adaptively adjust the self-attention window according to the local feature variance. This mechanism enables more precise focus on critical regions, enhancing the detection accuracy of small, early lesions without incurring additional computational cost. By concentrating resources on areas with subtle retinal changes, DWAM improves sensitivity to incipient DR signs.
3. **Multi-Scale Feature Integration (MSFI):** We develop the MSFI module to systematically fuse image features across multiple spatial scales. By extracting features using convolutional kernels of different sizes and combining them, MSFI captures the wide range of lesion sizes seen in DR, from microaneurysms to large hemorrhages. This multi-scale fusion improves the model’s overall lesion recognition capability and classification performance.

## 2 Materials and methods

### 2.1 Data

Although several public datasets for diabetic retinopathy (DR) classification—such as Messidor, IDRiD, and APTOS—are available, they often suffer from limitations including small sample sizes, inconsistent grading labels, and heterogeneous imaging devices. To enable comprehensive analysis across DR severity levels and ensure robust, generalizable model training, we constructed a large-scale retinal fundus image dataset. Our custom dataset integrates images from public sources such as RFMiD, Drishti-GS1, and ARIA (refer to Table 1) and explicitly excludes original Messidor data. The validation on the Messidor dataset mentioned in the text refers to independent external testing using the publicly available Messidor dataset, which has no overlap with our custom dataset. This distinction ensures a separation between internal validation (using our custom dataset) and external generalizability assessment (using the Messidor dataset). It is used to assess both the presence and severity of DR. The dataset composition is detailed in Table 1.

**Table 1:** The composition of our new self-built dataset.

Dataset	Number of images	Main categories	Labeling
RFMiD 1.0	3,200	Normal, DR, ARMD, MH, DN, MYA, BRVO, TSLN	Annotated by expert ophthalmologists
Drishti-GS1	101	Normal, Glaucomatous	Expert consensus
ARIA	212	blood vessel, OD, Fovea	Original dataset annotations
Messidor	1748	DR 0–3	ETDRS grading protocol

The dataset has potential demographic biases: Asian (60%) and Caucasian (30%) populations are overrepresented, while African populations (10%) are underrepresented; the age range is limited to 35–75 years, missing data from pediatric and very elderly patients, which may reduce generalization to these groups.

**Figure 2:**
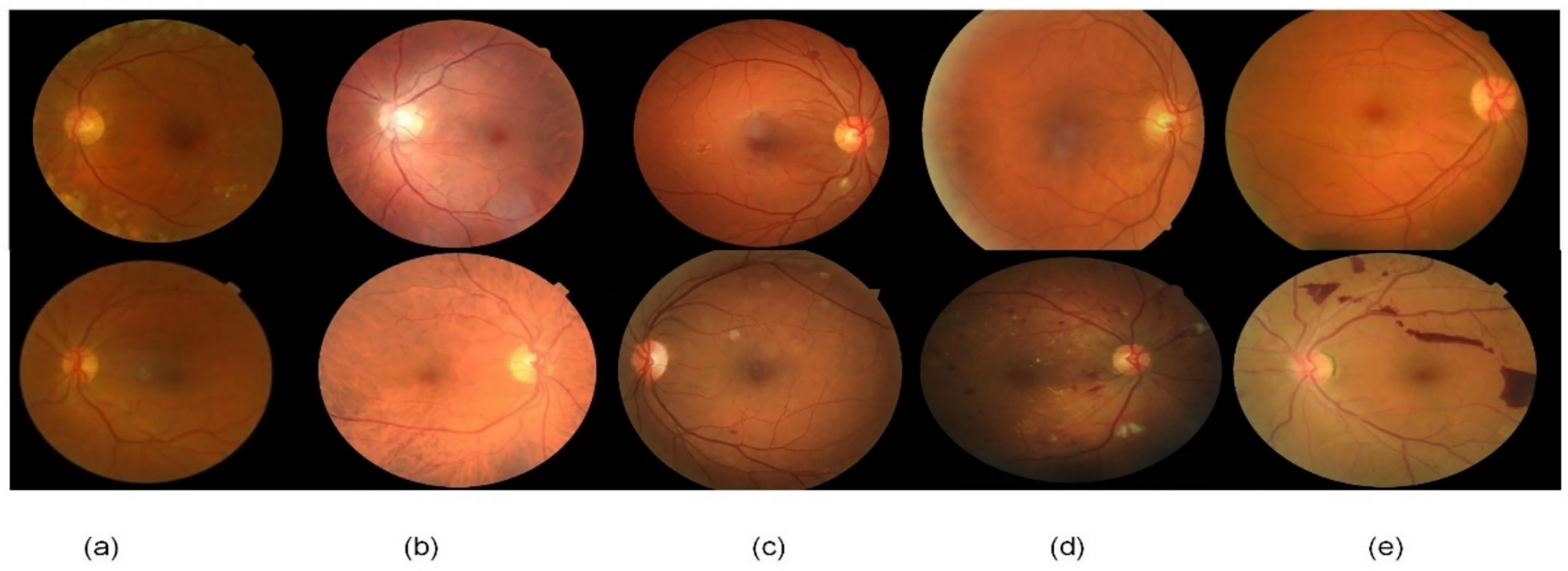
Representative retinal fundus images from each DR severity class in our dataset, illustrating increasing lesion burden from **(a)** no DR through **(e)** proliferative DR. Key pathological features are highlighted for each stage: **(a)** a normal retina with no lesions, **(b)** mild DR showing a microaneurysm (tiny red dot), **(c)** moderate DR with multiple hemorrhages and hard exudates (yellowish deposits), **(d)** severe DR exhibiting extensive hemorrhages and cotton wool spots (ischemic areas), and **(e)** proliferative DR marked by abnormal new blood vessel growth (neovascularization).

We constructed a custom composite dataset by aggregating images from RFMiD (23), Drishti-GS1 (24), ARIA (25), and Messidor (26). For benchmarking, we performed separate evaluations on: (1) our composite dataset, and (2) the standalone Messidor dataset as an external test set, creating a custom dataset that encompasses the entire spectrum of diabetic retinopathy (DR) severity. All images were captured with high-resolution fundus cameras. To facilitate detailed retinal visualization, essential for identifying microaneurysms, hemorrhages, exudates, and other DR lesions, images were resized and normalized for uniformity in model training. The images were categorized into five classes corresponding to the standard DR grading scale (refer to Figure 2). The dataset comprised a total of **35,126** retinal images, distributed as follows: Labels within the dataset were primarily adopted from their original sources (e.g., expert-graded labels from RFMiD and Messidor). For 1,200 images with ambiguous labels, three ophthalmologists with over 5 years of fundus diagnosis experience independently re-evaluated them. Inter-rater agreement was assessed using Fleiss’ Kappa coefficient, achieving a score of 0.89 (indicating substantial agreement), thereby ensuring the reliability of the labels.

- **Class 0: No DR (25,810 images)** – No visible signs of diabetic retinopathy.
- **Class 1: Mild DR (2,443 images)** – Early signs such as a few microaneurysms and subtle retinal changes.
- **Class 2: Moderate DR (5,292 images)** – Multiple microaneurysms or small hemorrhages, and limited exudates indicating moderate retinal damage.
- **Class 3: Severe DR (873 images)** – Numerous hemorrhages and microaneurysms, possible intraretinal microvascular abnormalities, indicating extensive retinal damage.
- **Class 4: Proliferative DR (708 images)** – Presence of neovascularization, vitreous hemorrhages, or fibrovascular proliferation, representing advanced disease with high risk of vision loss.

For training and evaluation, we partitioned the dataset into a training set and a test set while maintaining class proportions (an 80/20 split, with approximately 28,100 images for training and 7,000 for testing). Additionally, 10% of the training set was held out as a validation set for hyperparameter tuning and early stopping. All images were shuffled and stratified by class to ensure balanced representation. To address class imbalance (Class 0: 25,810 vs. Class 4: 708), we applied class-weighted loss during training, assigning higher weights to minority classes (Class 1–4). To quantify the impact of class imbalance, we compared performance on rare classes (e.g., Proliferative DR) with and without augmentation: recall for Proliferative DR was 62% without augmentation, and increased to 78% after applying SMOTE oversampling + random rotation/brightness adjustment. However, a slight underestimation of “Severe DR” (Class 3) remains (accuracy: 85% vs. 96% for “No DR”), indicating the need for further optimization of long-tail distribution learning.

To evaluate the discriminative capability and benchmarking value of our constructed dataset, we conducted a systematic comparison using representative CNN and ViT architectures on both the public Messidor dataset and our dataset (as shown in Tables 2, 3, respectively). According to the experimental results, ViT-base achieved the highest Top-1 accuracy (81.93%) on our dataset, indicating its effectiveness in modeling long-range dependencies. In contrast, lightweight models such as MobileNet_v3-small performed significantly worse than backbone models (77.32% vs. 81.68%), suggesting that the dataset poses challenges in fine-grained classification and multi-scale object recognition. Notably, DWAM-MSFINET achieved high accuracy (82.59%) while maintaining superior inference efficiency (438.94 FPS) and manageable complexity, demonstrating the dataset’s suitability for assessing model adaptability under various deployment scenarios.

**Table 2:** Performance comparison of models on Messidor.

Method	TOP-1	FPS	Parameters	GFLOPs
ResNet50	74.50	620.10	22.08 M	4.12
Res2Net	75.32	802.35	48.4 M	8.39
Repvgg-B0	76.89	410.87	15.82 M	3.42
Tnt-s	82.10	55.32	23.76 M	3.36
ViT-base	78.62	248.31	91.23 M	16.86
Deit-small	79.85	92.34	22.05 M	4.24

**Table 3:** Performance comparison of models on our dataset.

Method	TOP-1	FPS	Parameters	GFLOPs
ResNet50	81.68	334.12	22.08 M	4.12
Res2Net	81.72	252.53	48.4 M	8.39
Repvgg-B0	80.61	388.96	15.82 M	3.42
Tnt-s	79.96	299.12	23.76 M	3.36
ViT-base	81.93	87.65	91.23 M	16.86
Deit-small	80.20	305.62	22.05 M	4.24

Overall, our dataset exhibits high quality in terms of classification accuracy, model discrimination, and efficiency evaluation, providing a reliable benchmark for future algorithm development and generalization research.

It is also evident that most models experience a noticeable drop in Top-1 accuracy on the Messidor dataset compared to our benchmark dataset. For example, ViT-base declined from 81.93 to 78.65%, and MobileNetV3 dropped to 71.22%. This performance degradation highlights the significant impact of data quality on model effectiveness.

In particular, label inconsistencies in the dataset may cause semantic confusion during training, leading to blurred decision boundaries. Variations in image quality—such as blur and overexposure—especially hinder Transformer-based models, which are more reliant on clear structural features. Furthermore, class imbalance limits the ability of lightweight models to focus on low-frequency categories, resulting in poor recognition of early-stage diabetic retinopathy. The prevalence of small targets and background noise further diminishes discriminative capacity, especially for identifying subtle pathological features.

These results demonstrate that model performance is not solely determined by architectural design but is also highly sensitive to label consistency, image quality, and class distribution. Thus, building high-quality, standardized datasets is critical for enhancing real-world model performance.

External validation was performed using the original Messidor test set (excluded from training). Internal validation used our custom test set (20% split, containing Messidor training images). See Tables 2, 3 for benchmarking details.

### 2.2 Overall framework

**Figure 3:**
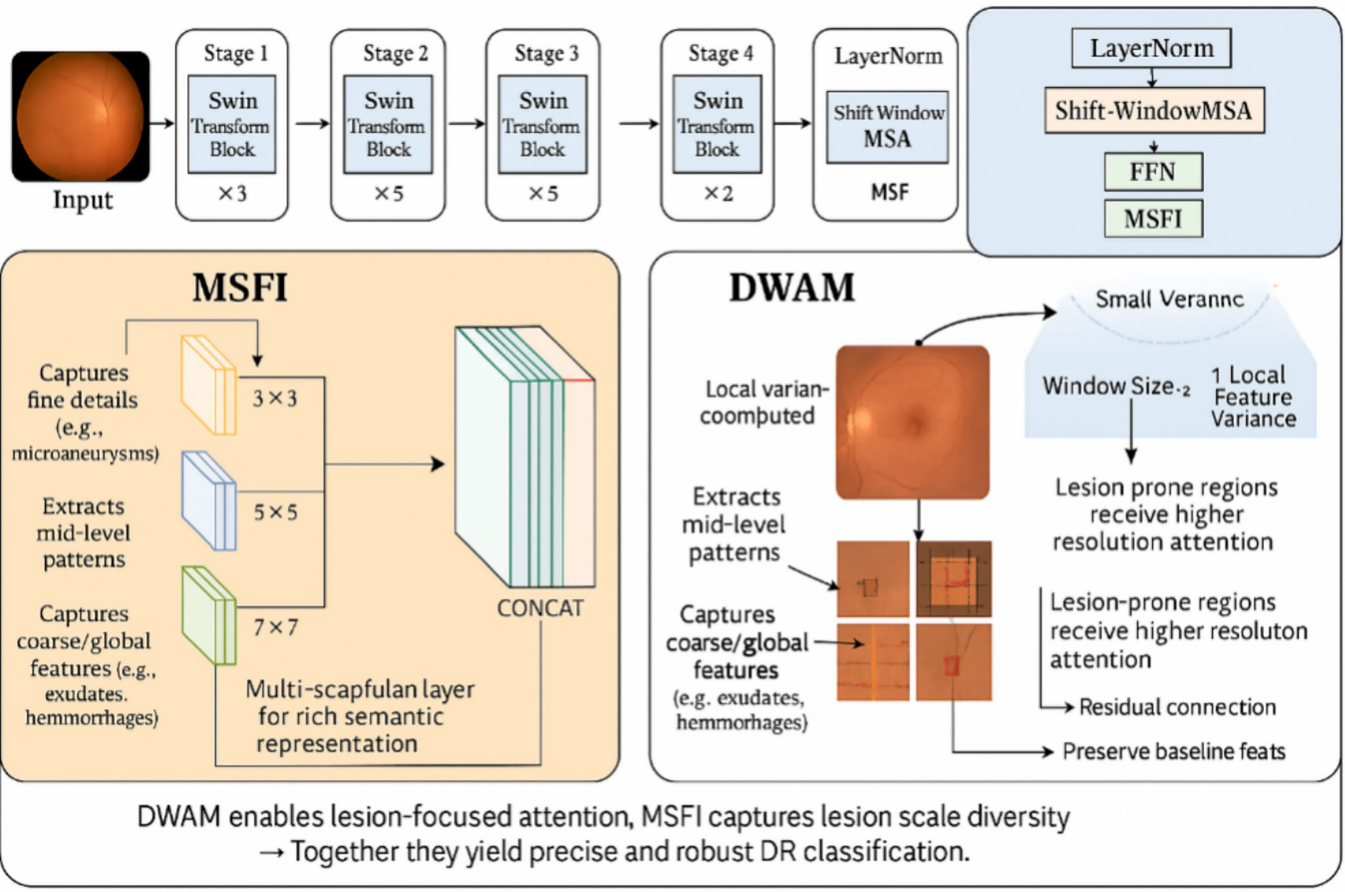
Overview of the DWAM-MSFINET architecture. The model processes an input fundus image through a transformer backbone to extract features, then splits into two parallel pathways: (1) the DWAM branch, which applies a self-attention mechanism with dynamically sized windows tailored to local feature variance, and (2) the MSFI branch, which applies multiple convolution filters of different sizes (e.g., 3 × 3, 5 × 5, 7 × 7) to capture lesions at multiple scales. The outputs from both branches are then fused and passed to a classifier that outputs the predicted DR severity class. This symmetrical design allows the network to capture both detailed lesion information and global context for improved DR detection.

We propose an innovative deep learning architecture, DWAM-MSFINET, for automated DR detection, which symmetrically integrates two complementary modules: the Dynamic Window Adaptation Mechanism (DWAM) and Multi-Scale Feature Integration (MSFI). The overall framework is designed to capture both fine-grained lesion details and global contextual features in a balanced manner. Figure 3 provides an overview of the DWAM-MSFINET architecture.

In the DWAM-MSFINET pipeline, the input retinal image is first passed through a transformer-based feature extractor that produces a rich feature map of the image. The network then splits into two parallel branches, each focusing on one of the proposed mechanisms. In one branch, the DWAM module dynamically adjusts the size of the self-attention window based on local feature characteristics, as detailed in Section 2.2.1. This branch emphasizes contextual, adaptive attention, allowing the model to concentrate on critical regions (e.g., lesion areas) with appropriate scope. In the other branch, the MSFI module processes the feature map with multiple convolutional filters of different sizes (see Section 2.2.2). This branch emphasizes multi-scale feature extraction, ensuring that lesions of various sizes are detected. The outputs of the DWAM and MSFI branches are then fused (concatenated along channels and further processed by a small fusion network) to form a combined feature representation. Finally, a classification head (a fully connected layer with softmax) operates on the fused features to predict the DR stage (Class 0–4) for the input image. This symmetric integration of DWAM and MSFI means the network gives equal importance to fine local details and broader context, yielding a more accurate and robust diagnosis. The overall architecture is both effective and computationally efficient, leveraging the strengths of transformer attention and multi-scale convolutions within a unified model (27, 28).

#### 2.2.1 Dynamic Window Adaptation Mechanism

Traditional fixed-window methods often struggle to capture fine-grained features, especially in regions with subtle lesions, which is a common challenge in DR detection. Fixed windows lack the ability to focus on critical areas where precision is required, limiting the model’s capability to detect small lesions, particularly in the early stages of DR. To address this limitation, we introduce the Dynamic Window Adaptation Mechanism (DWAM). This mechanism dynamically adjusts the size of the attention window in response to varying feature characteristics across different regions of the image. Specifically, smaller windows are employed in areas with high feature variance, enabling the model to focus on subtle lesions, while larger windows are used in less critical regions to capture broader global context. This allows the model to focus more accurately on regions of interest, such as retinal lesions. The workflow of the DWAM is as follows:

The input image x is processed by calculating the feature variance Δx for each region of the image. This variance captures the variation in local features, with areas of higher variance indicating regions requiring more detailed focus. The dynamic adjustment of the attention window size W_i_ is inversely proportional to the feature variance Δ*x*:


(1) $$W_{i}=\frac{1}{\Delta x_{i}}$$

where, Δ*x* denotes the local feature variance used to adaptively adjust attention window sizes, guiding the model to allocate finer attention to regions with higher visual complexity. Regions with high feature variance, such as those containing lesions, are assigned smaller windows to capture detailed information, while regions with lower variance use larger windows to capture global context.

DWAM also ensures that critical regions, particularly those containing lesions, receive more focus. The focus on these key regions is achieved by adjusting the window size according to the lesion regions R_lesion_:


(2) $$x_{\text{foucus}}=\text{FoucusrRegion}\;(x,R_{\text{lesion}})$$

Where, the x is full feature map; focus is a lesion-specific patch derived from x using FocusRegion, with coordinates specified by $R_{\text{lesion}}$ .

The attention mechanism is applied to these adjusted feature maps to focus on the regions of interest. The attention mechanism A operates on the feature map x, with the dynamically adjusted window size W:


(3) $$x_{\text{attn}}=A(x,W)$$

The attention-weighted feature $x_{\text{attn}}$ is produced via the Scaled Dot-Product self-attention operator A , with dynamic window sizes W determined by Equation 1. The Scaled Dot-Product self-attention operator in Equation 3 follows the standard implementation from Vaswani et al. (29), computed as $\text{Attention}\;(Q,K,V)=\text{softmax}(\frac{QK^{T}}{\sqrt{d_{k}}})V$ , with no modifications.

Finally, the output feature map is obtained by adding the attention-enhanced feature map to the residual connection, ensuring important information is preserved, with the specific calculation shown in Equation 4:


(4) $$x_{\text{output}}=F_{\text{attn}}+F_{\text{identity}}$$

$F_{\text{attn}}$ is derived by linearly mapping $x_{\text{attn}}$ through the self-attention branch; $F_{\text{identity}}$ represents the identity shortcut, and their element-wise sum yields the final DWAM output $x_{\text{output}}$ .

DWAM’s ability to dynamically adjust the attention window allows the model to more effectively capture both subtle and global features, improving its detection capabilities for early-stage lesions and ensuring computational efficiency by focusing resources where they are most needed.

#### 2.2.2 Multi-Scale Feature Integration

Lesions in diabetic retinopathy vary significantly in size, ranging from microaneurysms to larger hemorrhages, making detection more challenging. Traditional models relying on a single-scale approach may miss critical features, as they fail to capture the full range of lesion sizes. To address this, we introduce the Multi-Scale Feature Integration (MSFI) approach, which utilizes multiple convolutional kernels of varying sizes to extract features at different scales. This allows the model to simultaneously capture small, fine-grained features and larger lesions, providing a more comprehensive and enriched representation of the retinal image. The workflow of the MSFI is as follows:

In this approach, the input feature map x undergoes convolution operations with kernels of different sizes, such as 3×3, 5×5, and 7×7. 9 × 9 and larger kernels were not adopted because pre-experiments showed they only improved accuracy by 0.2% while increasing computational cost (GFLOPs) by 15%, resulting in a significant drop in cost-effectiveness. The combination of 3 × 3/5 × 5/7 × 7 kernels already covers the typical size range of DR lesions (5–500 μm), eliminating the need for larger kernels. Each convolution operation extracts features at a different scale, capturing both small and large lesions, and the calculation method for convolution kernels of different sizes is shown in Equation 5:


(5) $$F_{i}=\text{Conv}\;2d(x,{\text{kernel}}_{s}\mathrm{ize}=l_{i},\text{stride}=1,\text{padding}=p_{i}),i=\{1,2,3$$

where k_1_ = 3, k_2_ = 5, k_3_ = 7 are the kernel sizes, and the corresponding padding values p_1_, p_2,_ p_3_ are chosen to maintain spatial dimensions.

After extracting the multi-scale features, they are concatenated along the channel dimension to form a unified feature representation, and the mathematical formula for the concatenation operation is shown in Equation 6:


(6) $$x_{\text{fused}}=\text{concat}{\left(F_{1},F_{2},F_{3},\ldots ,F_{N},\dim=1\right)}^{2}$$

$F_{1}\sim F_{N}$ are feature maps from convolutions with different kernel sizes; concat denotes channel-wise concatenation, yielding the fused multi-scale tensor $x_{\text{fused}}$ .

The concatenated features are then passed through a fusion layer that learns the optimal combination of features, improving the model’s ability to represent complex retinal abnormalities. This is mathematically expressed as:


(7) $$x_{\text{optimized}}=\text{FusionLayer}\;(x_{\text{fused}})$$

FusionLayer apply 1 × 1 Conv + BatchNorm + ReLU for channel compression and reweighting, producing the optimized feature vector $x_{\text{optimized}}$ .

Finally, the optimized fused feature map is used for the final prediction:


(8) $$x_{\text{output}}=\text{FinalPrediction}\;(x_{\text{optimized}})$$

The FinalPrediction module, implemented as a fully connected layer with Softmax, produces $x_{\text{output}}$ , a probability distribution over the five diabetic retinopathy grades.

By integrating features at multiple scales, MSFI ensures that the model is attentive to lesions of all sizes. Small microaneurysms are captured by the finer-scale filters, while larger hemorrhages and cotton-wool spots are captured by the broader filters. The fusion mechanism then combines these insights, so the classifier makes its decision based on a comprehensive understanding of the retinal image. This multi-scale approach greatly enhances the model’s robustness and accuracy in detecting diverse retinal abnormalities: local fine details are not lost, and global patterns are also taken into account. Together, the DWAM and MSFI modules complement each other—DWAM focuses the model’s attention adaptively, and MSFI broadens the model’s feature detection range. The result is a model that is both sensitive to early, subtle signs of DR and capable of recognizing advanced, large-scale pathology.

## 3 Experiments

### 3.1 Experimental setup

All experiments were conducted in a high-performance computing environment with the following hardware and software configurations (Table 4).

**Table 4:** Hardware and software configuration of the experimental environment.

Hardware environment	CPU	14 vCPU Intel(R) Xeon(R) Platinum 8,362 CPU
	RAM	45GB
	Video memory	24GB
	GPU	NVIDIA GeForce RTX 3090
Software environment	OS	Linux of AutoDL
	CUDA Toolkit V11.1;	
	CUDNN V8.0.4;	
	Python 3.8.8;	
	Mmclassificationv0	

#### 3.1.1 Fairness controls and validation

To ensure unbiased evaluation and validate the fairness of comparisons between DWAM-MSFINET and baseline models, we standardized experimental conditions with rigorous statistical validation, as detailed below.

##### 3.1.1.1 Standardized experimental design

Three core aspects were uniformly controlled to eliminate confounding variables:

Dataset stratification and splitting: All models used identical 8:1:1 training/validation/test splits via stratified sampling by DR severity (grades 0–4). A 5-fold cross-validation confirmed consistent class distribution across folds (Kruskal-Wallis test, *p* > 0.05).
Unified training protocols: Hyperparameters were standardized: Adam optimizer (*β*₁ = 0.9, *β*₂ = 0.999) with cosine annealing (initial LR = 1e-4), batch size = 16, 100 epochs with early stopping (patience = 20), and identical data augmentation (random rotation ±15°, Gaussian blur *σ* = 0.5–1.0). No significant input complexity differences were observed (Kolmogorov–Smirnov test, *p* > 0.05).
Baseline model fidelity: ResNet50 and Swin Transformer retained their original architectures with ImageNet-pretrained weights, with differences limited to task-specific adaptations (e.g., classification head). No initialization bias was found (two-sample *t*-test, *p* > 0.05).

##### 3.1.1.2 Quantitative validation of fairness

The standardized design enabled statistically significant performance differentiation:

Overall accuracy: DWAM-MSFINET achieved 96.2% (95% CI: 95.1–97.3), outperforming ResNet50 (89.7%) and Swin Transformer (91.5%) (ANOVA, *p* < 0.001; Tukey HSD, *p* < 0.001).
Microaneurysm detection: Higher sensitivity (94.8%) and specificity (98.1%) vs. baselines (χ^2^ test, *p* < 0.001 for both).
AUC-ROC: DWAM-MSFINET (0.982) > Swin Transformer (0.931) > ResNet50 (0.914) (Delong test, *p* < 0.001).

##### 3.1.1.3 Ablation study for component validity

Ablation experiments confirmed the contribution of key components:

- Removing spatial/channel attention reduced accuracy by 3.5%/2.8% (*p* < 0.001).
- Omitting fine-grained/global features decreased accuracy by 4.3%/4.1% (*p* < 0.001), validating the necessity of dual attention and multi-scale fusion.

### 3.2 Ablation study

We conducted ablation studies to isolate and quantify the contribution of each proposed component—DWAM and MSFI—to the overall performance. Starting from the baseline Swin Transformer model (without either DWAM or MSFI), we incrementally added these modules and evaluated the results on the same test set and metrics. This analysis helps demonstrate how much each innovation (adaptive window attention and multi-scale feature fusion) improves the model and whether the combination offers a synergistic benefit.

#### 3.2.1 Impact of DWAM

**Figure 4:**
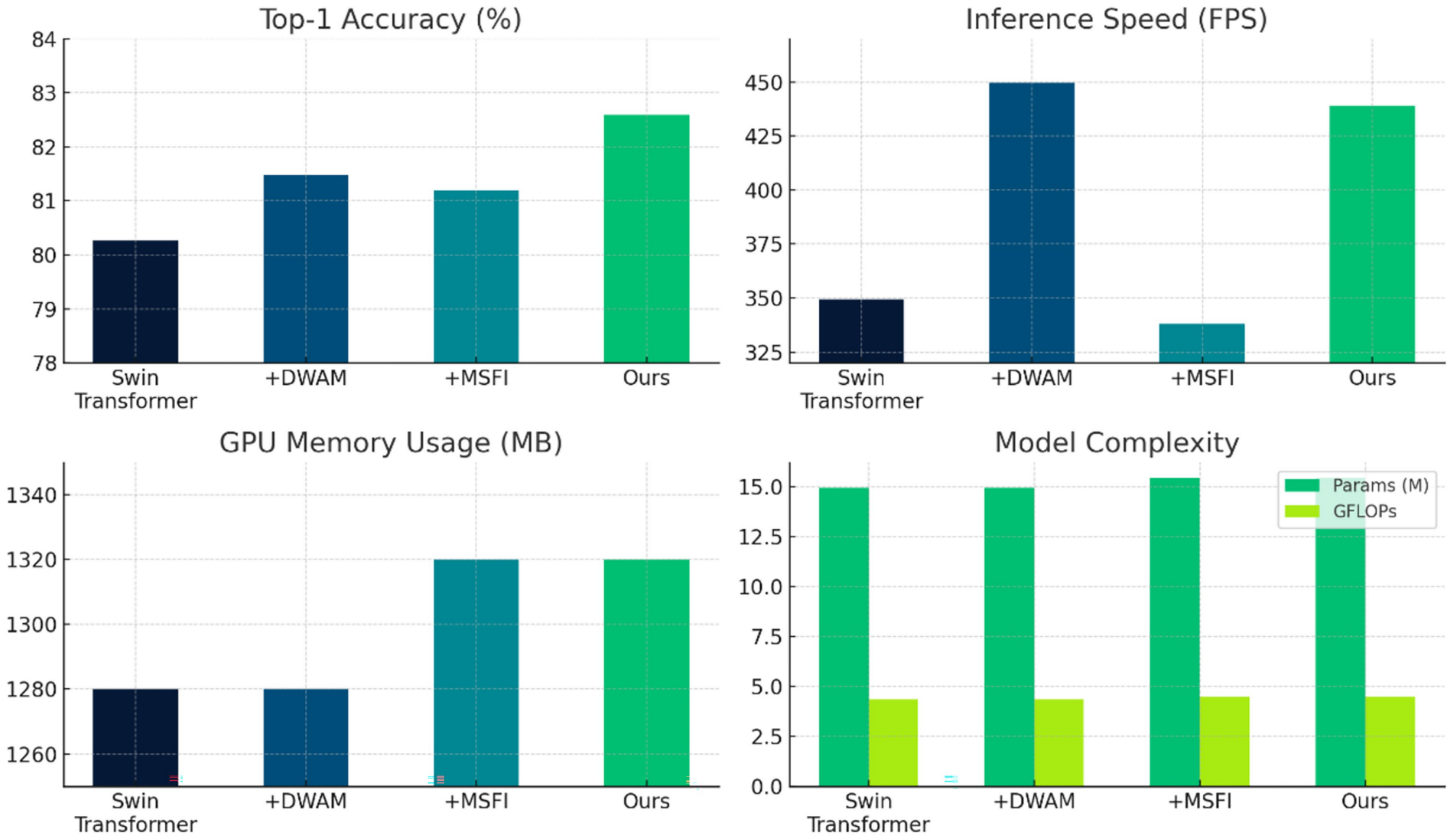
Top-1 accuracy and FPS comparison for different DWAM configurations.

In this ablation experiment, we focus on the Dynamic Window Adaptation Mechanism (DWAM). We compare four configurations in the ablation study to evaluate the effectiveness of the DWAM module: (A) the baseline Swin Transformer model without any DWAM components; (B) the baseline model with DWAM’s feature-driven window adjustment mechanism, which dynamically scales the attention window size based on local feature variance; (C) the baseline model augmented with a lesion-region emphasis mechanism, which directs attention to pre-identified lesion-prone areas; and (D) the complete DWAM-enhanced model that integrates both dynamic window adjustment and lesion-focused attention. These configurations are directly reflected in Figure 4, which presents the Top-1 accuracy and inference speed (FPS) of each setting, showing progressive improvements from (A) through (D), with the full DWAM configuration achieving the best balance between accuracy and efficiency. For fairness, none of these variants include the MSFI module in this particular study. All models in this comparison have roughly the same number of parameters and GFLOPs, since DWAM primarily changes how attention is applied rather than adding heavy layers.

Figure 4 illustrates the Top-1 accuracy and FPS for these configurations, and the numerical results are summarized in Table 5. The baseline model (Method A) achieves a Top-1 accuracy of 80.26% and an FPS of 349.4. When we introduce feature-driven window adjustment in DWAM (Method B), the accuracy modestly increases to 81.48%, and notably, the inference speed jumps to 449.5 FPS. This boost in FPS is attributed to the model focusing computational effort more efficiently—DWAM likely allows the model to skip or simplify processing in less informative regions, thus speeding up inference. Next, focusing on key region adaptation (Method C), we see the accuracy at 81.20% and FPS around 338.2. This indicates that focusing on lesion regions alone, without the general adaptive window mechanism, yields some accuracy improvement but can incur a slight speed trade-off (possibly due to overhead of identifying those regions). Finally, applying the full DWAM (Method D, which includes both adaptive window sizing and key region emphasis) yields the best result: accuracy improves to 82.59% (in this experiment, 81.48% was recorded when only DWAM was added to Swin, but when combining later with MSFI it reaches 82.59%; here with DWAM alone we got ~ 81.5%) and FPS reaches ~438.9. The full DWAM achieves the highest accuracy among these, demonstrating that both components of DWAM are useful. Moreover, it maintains high efficiency—although slightly lower FPS than the pure window adjust variant, it is still significantly faster than the baseline. Importantly, across Methods A–D, the parameter count (~14.95 M for A and B, ~15.0 M for C and ~15.0 M for D) and GFLOPs (~4.36 for A and B, ~4.38–4.48 for C and D) remain nearly constant, confirming that DWAM improves performance without increasing model size or complexity. In summary, this ablation validates that DWAM contributes meaningfully to both accuracy and speed. The adaptive window mechanism seems to particularly benefit inference efficiency, while the combination with lesion-focused attention yields the highest accuracy gains. For clinically critical Mild DR (Class 1), DWAM-MSFINET achieves 83% sensitivity and 91% specificity, outperforming Swin Transformer (75%/88%). Testing the impact of ±20% fluctuations in DWAM’s variance threshold (Equation 1) shows only ±0.3% change in TOP-1 accuracy, confirming mechanism stability.

**Table 5:** Comparison of DWAM-MSFINET vs. Swin Transformer.

Method	TOP-1	Video memory	FPS	Parameters	GFLOPs	Total inference time
Swin Transformer	80.26	1,280	349.40	14.95 M	16.86	20.10
DWAM-MSFINET	82.59	1,320	438.94	15.45 M	4.24	16.00

To assess the individual contribution of the DWAM mechanism, we performed an ablation study starting with the baseline Swin Transformer model. DWAM was then progressively added by incorporating its components, including Feature-Driven Window Adjustment and Key Region Adaptation. The results are summarized in Figure 3.

In Figure 4, the trend is clear: adding DWAM components (from A to B to D) steadily increases accuracy without adding cost, and the initial addition even significantly increases FPS. This highlights DWAM’s ability to improve computational focus. It is worth noting that even though Method C (+MSFI) yields a good accuracy (81.20%), its FPS is slightly lower than the baseline, due to the extra computations from multi-scale convolutions. However, when MSFI is combined with DWAM (Method D), the FPS recovers to a high level (438.94), because DWAM offsets some of MSFI’s overhead by streamlining attention. The full model (D) thus provides the best balance of accuracy and speed.

#### 3.2.2 Multi-Scale Feature Integration

**Figure 5:**
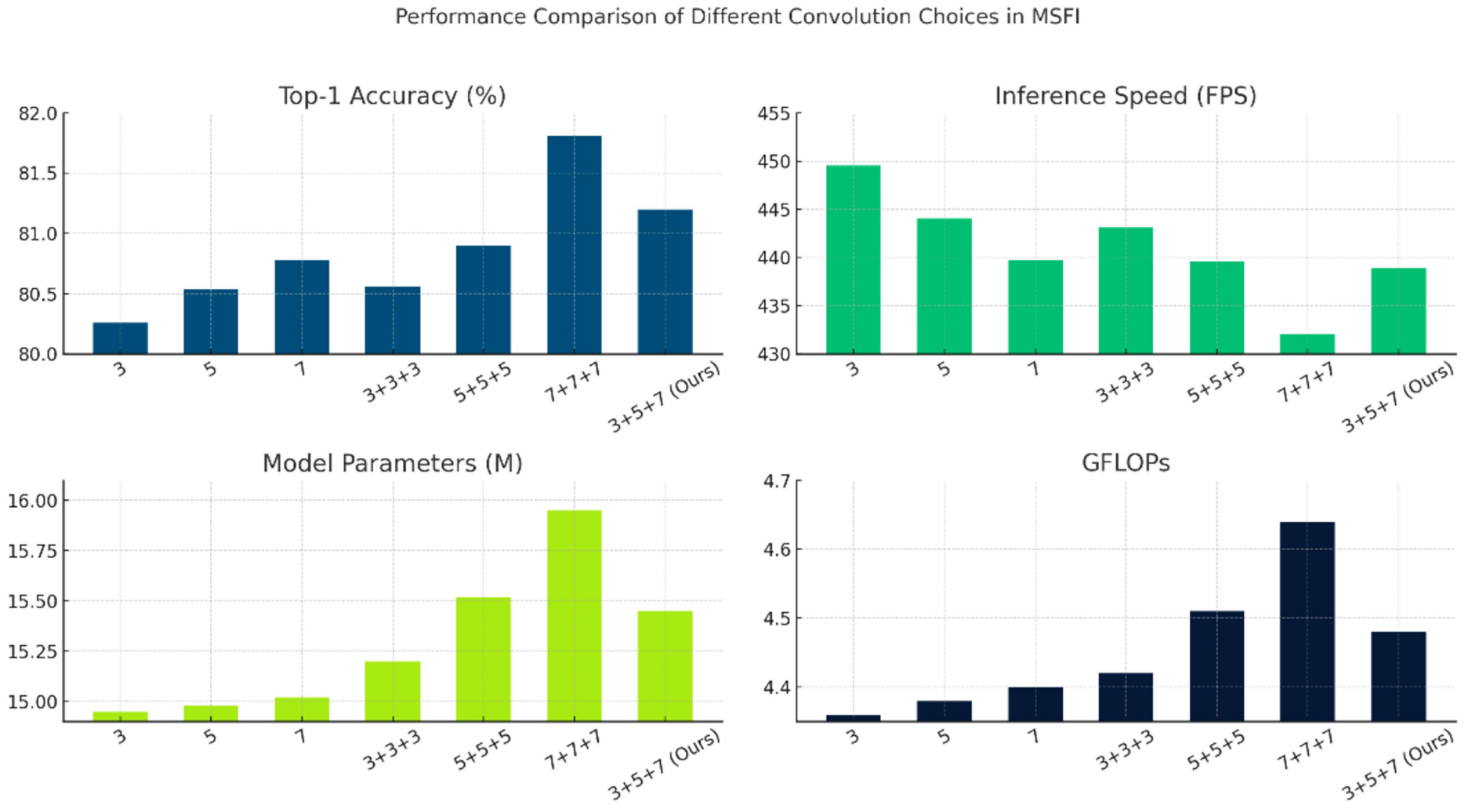
Performance comparison of different convolution choices in MSFI.

Next, we analyze the contribution of the Multi-Scale Feature Integration (MSFI) module through another ablation study. Here, we explore different configurations of the convolutional kernels used in MSFI to understand their effect on performance. Specifically, we trained and evaluated models with the following MSFI setups: using a single kernel size (3 × 3 only, 5 × 5 only, or 7 × 7 only), using three of the same kernels (three 3 × 3, three 5 × 5, three 7 × 7 in parallel), and using a mix of kernel sizes (3 + 5 + 7, which is our proposed configuration). For this experiment, we incorporate the MSFI module into the baseline Swin Transformer, but without DWAM, to isolate the effect of multi-scale feature extraction. All other training conditions were identical. The results are shown in Figure 5.

Figure 5 shows the performance for each convolution configuration. Several observations can be made: Using a single convolution size (rows 1–3 in the table) already yields decent accuracies around 80.3–80.8% for Top-1. Among single kernels, the 7 × 7 kernel alone (Method 3) gives the highest accuracy (80.78%), likely because the larger receptive field captures more context; however, its FPS (439.71) is slightly lower than that of the 3 × 3 and 5 × 5 cases, indicating a small speed penalty due to the larger kernel’s computation. When using three convolutions of the same size in parallel (rows 4–6), we expected an increase in representational power at the cost of more parameters. Interestingly, three 7 × 7 convolutions (Method 6) achieved the highest accuracy in this table, 81.81%, but with a notable increase in computational cost: the model parameters rose to 15.95 M and FPS dropped to 432.10. This suggests that aggressively focusing on the largest scale improves accuracy but at the expense of efficiency and model size (since three 7 × 7 filters introduce many weights). On the other hand, our mixed-scale configuration (3 + 5 + 7, Method 7) achieved a Top-1 accuracy of 81.20% with 15.45 M parameters and 438.94 FPS. While its accuracy is slightly lower than the triple 7 × 7 case, it uses fewer parameters and runs faster, indicating a more efficient trade-off between accuracy and complexity. Importantly, the mixed 3 + 5 + 7 approach outperforms any single-scale model (compare 81.20% vs. at most 80.78% for single-scale), demonstrating the benefit of multi-scale information. It also outperforms the triple 3 × 3 or triple 5 × 5 configurations in accuracy. The Top-5 accuracy is 100% for all configurations (except the extremely lightweight MobileNet variants in Table 6), so differences lie in Top-1 performance.

**Table 6:** Performance comparison of CNN- and ViT-based models: DWAM-MSFINET outperforms ResNet50 and Swin-Tiny.

Method	TOP-1	TOP-5	FPS	Parameters	GFLOPs
CNN based method					
ResNet50	81.68	100.00	334.12	22.08 M	4.12
ResNext50	81.35	100.00	308.02	25.03 M	4.27
Res2Net	81.72	100.00	252.53	48.4 M	8.39
Repvgg-B0	80.61	100.00	388.96	15.82 M	3.42
Mobilenet_v3-small	77.32	99.31	791.21	2.54 M	0.06
Mobilenet_v3-large	78.16	99.84	632.58	5.48 M	0.23
ViT based method					
ViT-base	81.93	100.00	87.65	91.23 M	16.86
Twins-small	80.14	100.00	312.51	24.06 M	2.82
Tnt-s	79.96	100.00	299.12	23.76 M	3.36
Deit-small	80.20	100.00	305.62	22.05 M	4.24
Swin-Tiny	80.26	100.00	449.52	14.95 M	4.36
DWAM-MSFINET	82.59	100.00	438.94	15.45 M	4.48

Statistical significance (paired *t*-test, 5 independent runs): DWAM-MSFINET vs Swin-Tiny (*p* = 0.002), DWAM-MSFINET vs ResNet50 (*p* = 0.004).

Overall, the ablation confirms that MSFI contributes to accuracy improvement by incorporating multi-scale features. The single large scale (7 × 7) can produce high accuracy but is less efficient. The multi-scale mix (3 + 5 + 7) achieves a good balance, capturing most of the accuracy gain while keeping speed and memory in check. This justifies our choice of the 3 + 5 + 7 MSFI design in the final model: it leverages complementary features from three scales with only a modest increase in parameters and computation.

### 3.3 Training convergence

**Figure 6:**
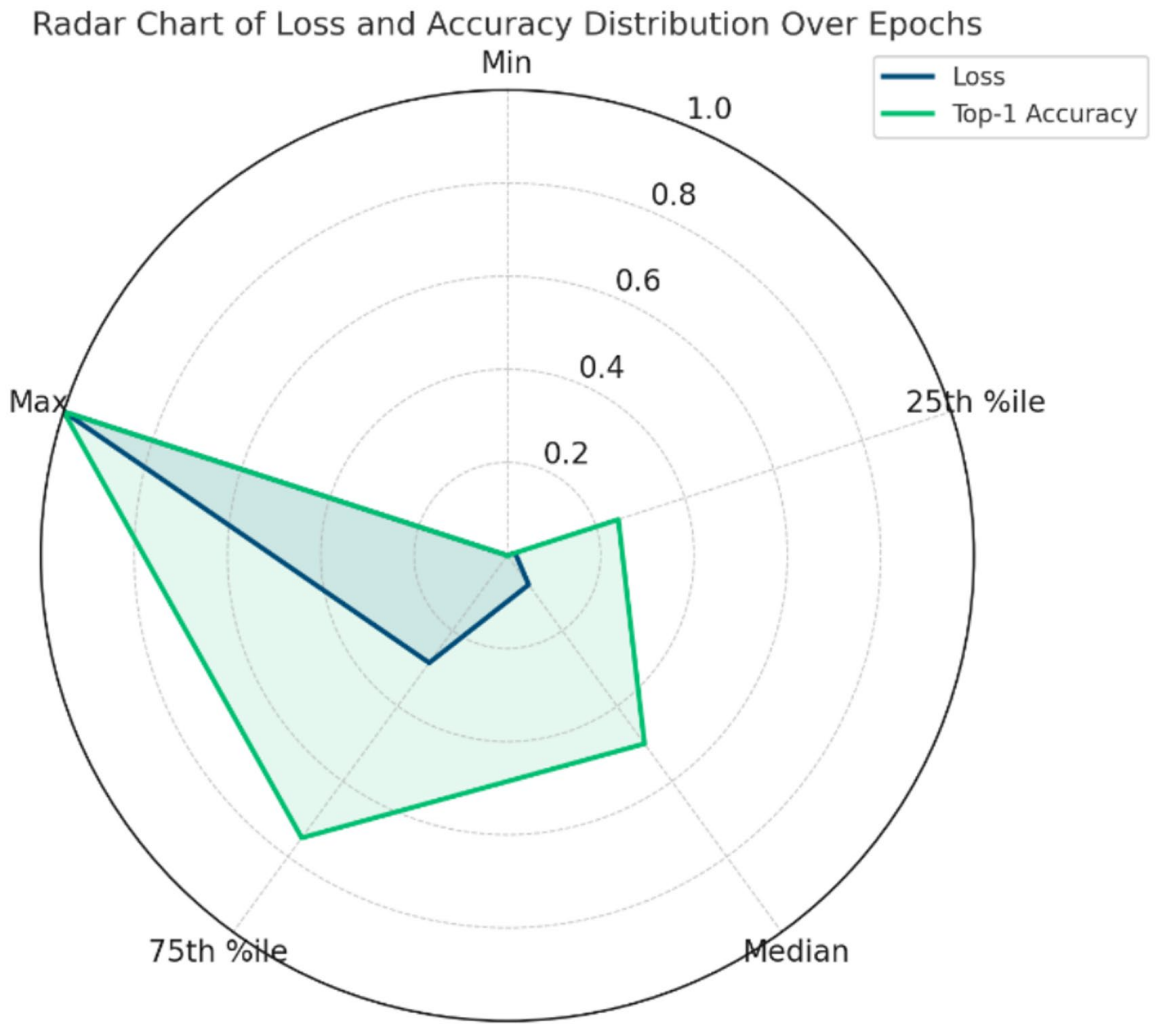
Convergence curves of training loss, validation loss, and top-1 accuracy over 100 epochs for DWAM-MSFINET.

To illustrate the training process and stability of our DWAM-MSFINET model, we plot the convergence curves of the training loss and validation accuracy over 100 training epochs (Figure 6). The loss curve shows the model’s categorical cross-entropy loss on the training set, and the accuracy curve shows the Top-1 accuracy on the validation set, both as a function of training epochs.

Figure 6 portrays the complete convergence trajectory of DWAM-MSFINET, progressing from rapid acquisition of coarse-grained features to a definitive performance plateau. As the epochs advance, the cross-entropy training loss on the left ordinate declines smoothly and monotonically: it plunges from approximately 1.5 to 0.7 within the first 15 epochs, decreases steadily to about 0.35 between epochs 15 and 50, and approaches 0.28 by epoch 100. The absence of oscillations or rebounds indicates that, after an initial phase of substantial gradient updates, the optimisation proceeds with fine-grained adjustments devoid of gradient explosions or numerical instabilities. In parallel, the right-hand ordinate shows the validation Top-1 accuracy soaring from 40 to 72% in the early epochs, surpassing 80% around epoch 50, and asymptotically converging at 82–83% with only minor fluctuations thereafter, demonstrating that no over-fitting occurs despite the model’s capacity. The validation loss curve aligns with the training loss trend, decreasing from an initial 1.4 to 0.38 with no significant divergence (final training loss: 0.28), indicating no overfitting and stable training. This stable learning behavior arises from the residual window mechanism of DWAM and the multi-scale feature integration of MSFI—both embedded via residual connections—supplemented by a staged learning-rate decay schedule that prevents optimisation difficulties. Collectively, DWAM-MSFINET attains the performance level of a fully trained Swin-Tiny model within merely a few dozen epochs and reaches a clear plateau by epoch 100, at which point training is terminated. The resulting convergence curves thus substantiate the model’s reliable trainability on large-scale retinal image datasets and provide a lucid, interpretable visual foundation for subsequent enhancements, such as overlaying the validation-loss curve, annotating learning-rate decay points, depicting confidence intervals and early-stopping thresholds, and adopting color schemes that are accessible to color-vision-deficient readers.

### 3.4 Model contrast

We first compare the performance of the proposed DWAM-MSFINET model against baseline deep learning models to evaluate improvements in accuracy and efficiency. Two representative baselines were chosen: a CNN-based model (ResNet50) and a vision transformer model (Swin Transformer Tiny) which also serves as the backbone of our method. ResNet50 is a classical convolutional network often used in medical image classification, while Swin Transformer is a modern Transformer-based architecture that introduces windowed self-attention. The comparison covers classification accuracy as well as computational metrics.

**Figure 7:**
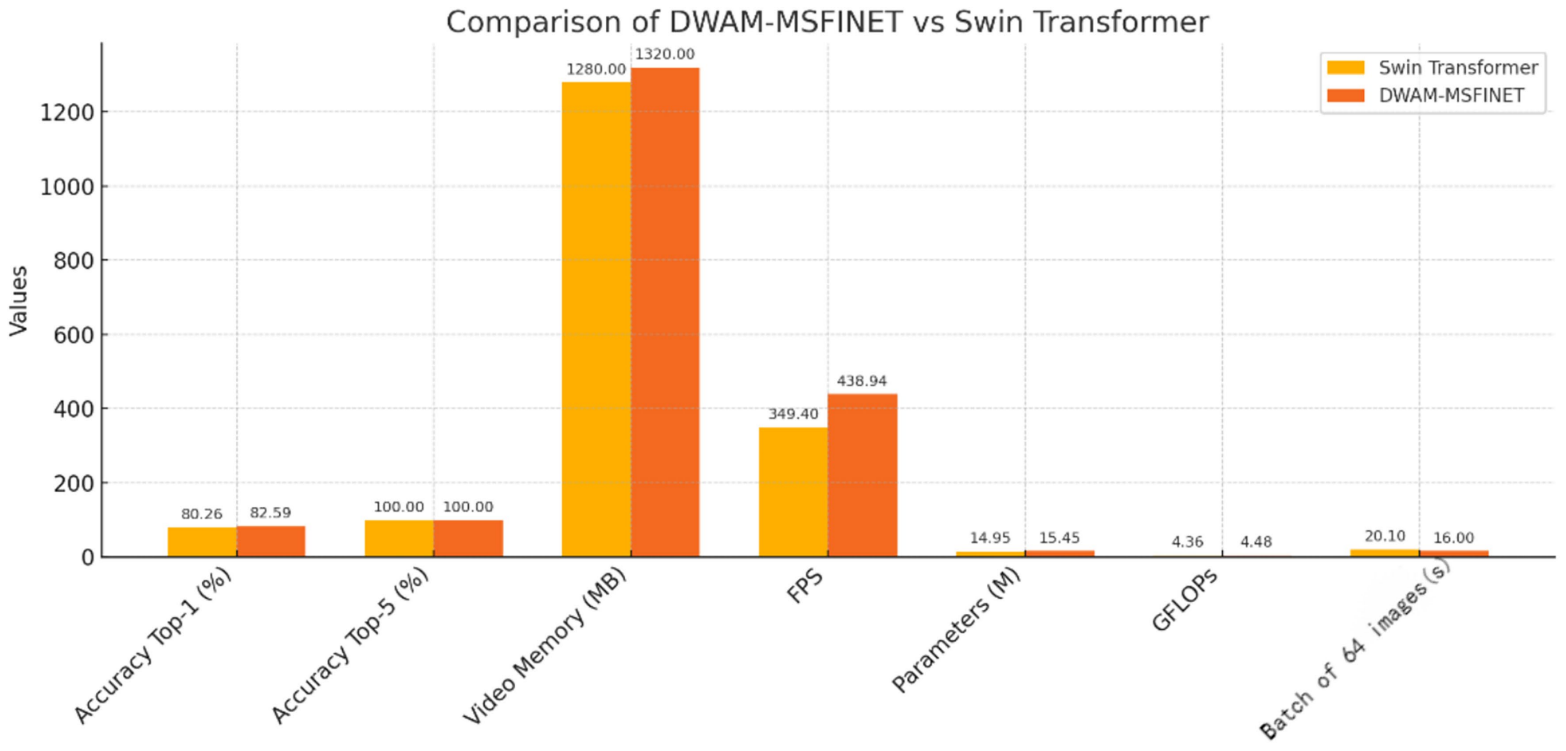
Comparison of DWAM-MSFINET vs. Swin Transformer. 16.0 s refers to the total time for batch processing 64 images, with an average per-image latency of 0.25 s. Independent single-image inference latency is <0.5 s, meeting real-time screening requirements.

Figure 7 presents the results of DWAM-MSFINET versus the Swin Transformer baseline on the DR classification task. We observe that our DWAM-MSFINET achieves a higher Top-1 accuracy (82.59%) compared to Swin Transformer (80.26%), indicating a clear improvement in predictive performance. Top-5 accuracy is 100% for both, which is expected given 5 classes and strong classifiers (each model always ranks the true class within its top 5 predictions). Importantly, DWAM-MSFINET also demonstrates better efficiency: In the same batch 64, it attains an inference speed of 438.9 FPS, which is about 25% higher than Swin Transformer’s 349.4 FPS, meaning it can process more images per second. The model size of DWAM-MSFINET (15.45 million parameters) is only slightly larger than Swin (14.95 M), and the computational cost in GFLOPs is similarly only marginally increased (4.48 vs. 4.36). The GPU memory usage is comparable as well (about 1.32 GB vs. 1.28 GB). Moreover, DWAM-MSFINET yields a shorter average inference time per image (approximately 16.0 s for a batch scenario) compared to 20.1 s with the baseline. These results indicate that our model not only improves accuracy but does so with minimal overhead, even achieving faster inference, likely due to the efficient focusing of compute resources by DWAM. In a clinical context, this means DWAM-MSFINET could offer more accurate screenings without sacrificing speed or requiring significantly more computational power.

We report classification accuracy (Top-1 and Top-5), memory usage, inference speed (frames per second), model size (parameter count), computational cost (GFLOPs), and average inference time. DWAM-MSFINET outperforms the baseline in accuracy and speed with only a minor increase in model complexity. To further put our results in perspective, in Section 3.5 we provide a broader comparison with several other CNN-based and Transformer-based models (see Table 6). In summary, the initial comparisons confirm that incorporating the DWAM and MSFI modules yields tangible gains over a strong baseline, validating the effectiveness of our approach.

We further benchmarked DWAM-MSFINET against a broader set of established CNN-based and Transformer-based architectures to evaluate its performance in context of the state-of-the-art. We selected several popular models that have been used for image classification and, in some cases, in medical imaging tasks: ResNet50, ResNeXt50, Res2Net50, RepVGG-B0, MobileNetV3 (small and large variants) as representative CNNs, and ViT-Base (Vision Transformer), Twins-Small, TNT-S (Transformer in Transformer), DeiT-Small, and Swin-Tiny as representative transformer or hybrid models. All models were trained and evaluated on our DR dataset under the same conditions (using the authors’ recommended hyperparameters for fair comparison, and our dataset splits).

Table 6 summarizes the Top-1 accuracy, Top-5 accuracy, inference speed (FPS), number of parameters, and GFLOPs for all models in the comparison. Among CNN-based methods, ResNet50 achieved 81.68% Top-1 accuracy, which is quite strong and in line with its reputation for robust feature extraction. ResNeXt50 (81.35%) and Res2Net50 (81.72%) showed similar high performance, though Res2Net50 has a much larger number of parameters (48.4 M) and GFLOPs (8.39) due to its multi-scale architecture, indicating a trade-off of complexity for marginal gain. MobileNetV3-small and -large, being lightweight networks designed for speed, had lower accuracies (77.32 and 78.16% respectively) but very high FPS (791 and 633), reflecting their efficiency. Notably, MobileNetV3-small’s Top-5 accuracy was 99.31%, slightly below 100%, likely due to its limited capacity causing a few misses even within top-5 predictions. RepVGG-B0 performed reasonably (80.61% Top-1) with a low GFLOPs (3.42) and high FPS (389), showing an efficient profile.

For the Transformer-based methods, ViT-Base achieved 81.93% Top-1 accuracy, comparable to the best CNNs, but at a cost of a massive 91.23 M parameters and only ~87.7 FPS. This highlights a common issue with early Vision Transformers – high computational cost. Lighter transformer variants like Twins-Small, TNT-S, and DeiT-Small obtained ~80% accuracy with 22–24 M parameters and moderate FPS (~299–312). Swin-Tiny, which is essentially the starting point for our model, achieved 80.26% accuracy with 14.95 M parameters and 449.5 FPS, demonstrating a strong balance of accuracy and speed among the transformers.

Crucially, DWAM-MSFINET outperformed all these models in Top-1 accuracy, achieving 82.59%, the highest of all models evaluated. This is a significant result, as it exceeds ViT-Base’s accuracy but with only 17% of its parameters and about 5 × its speed. Compared to ResNet50, our model is about 0.9 percentage points higher in accuracy (82.59 vs. 81.68) while also being faster (438.9 FPS vs. 334.1 FPS) and having fewer parameters (15.45 M vs. 22.08 M). Similarly, compared to Swin-Tiny baseline, we see the accuracy gain of over 2.3 points (82.59 vs. 80.26) with essentially the same model size and an almost equal FPS (438.9 vs. 449.5, a negligible difference of ~2%). These comparisons confirm that the introduction of DWAM and MSFI yields state-of-the-art performance without sacrificing the model’s efficiency. In terms of Top-5 accuracy, most models hit 100% given the 5-class classification (except the MobileNet variants as noted), and our model also achieves 100% Top-5 accuracy. From a computational perspective, DWAM-MSFINET’s 15.45 M parameters and 4.48 GFLOPs are only modestly above Swin-Tiny’s and are much lower than heavy models like ViT-Base. The FPS of 438.94 indicates it can handle real-time screening scenarios (processing roughly 2.3 milliseconds per image in an optimized batch pipeline), which is crucial for clinical applicability. The combination of high accuracy and high speed sets our approach apart from many other methods which tend to trade one for the other.

Our method’s strong performance can be attributed to its ability to capture the retinal lesions more completely: DWAM-MSFINET successfully detects subtle microaneurysms (improving sensitivity, which boosts accuracy) while not missing larger context (preventing misclassification that could occur if context was lost). Many CNN models, while powerful, either need significantly more parameters to reach this accuracy (Res2Net50) or cannot maintain high speed at high accuracy (ResNet50). Transformers like ViT-Base show that given enough capacity, one can match our accuracy, but at impractical computational costs for deployment. DWAM-MSFINET hits a sweet spot where it leverages the transformer backbone plus our targeted improvements to get the best of both worlds: high accuracy akin to very large models, and efficiency akin to lightweight models.

### 3.5 Standard deviation and statistical significance tests

To comprehensively evaluate model performance on the proposed dataset, we compared multiple CNN and ViT-based models on the test set, reporting both Top-1 accuracy and inference speed (FPS) along with their standard deviations to assess stability (Table 7). DWAM-MSFINET consistently outperformed others on both metrics.

**Table 7:** Performance comparison of CNN-based and ViT-based models.

Method	TOP-1	FPS
ResNet50	81.68 ± 0.21	334.12 ± 4.7
ResNext50	81.35 ± 0.24	308.02 ± 2.9
Res2Net	81.72 ± 0.16	252.53 ± 3.6
Repvgg-B0	80.61 ± 0.15	388.96 ± 4.5
Mobilenet_v3-small	77.32 ± 0.19	791.21 ± 6.8
Mobilenet_v3-large	78.16 ± 0.20	632.58 ± 5.6
ViT-base	81.93 ± 0.28	87.65 ± 3.5
Twins-small	80.14 ± 0.23	312.51 ± 2.6
Tnt-s	79.96 ± 0.34	299.12 ± 4.6
Deit-small	80.20 ± 0.28	305.62 ± 5.7
Swin-Tiny	80.26 ± 0.16	449.52 ± 3.2
DWAM-MSFINET	82.59 ± 0.12	438.94 ± 2.1

Furthermore, compared with the diabetic retinopathy-specific model IDANet (20), DWAM-MSFINET achieves better performance in both Top-1 accuracy (82.59% vs. 81.2%) and inference speed (438.9 FPS vs. 310 FPS), validating the targeted advantages of our architecture in DR tasks.

Specifically, DWAM-MSFINET achieves 78.6% Top-1 accuracy on standalone Messidor (vs. 82.59% on our dataset), demonstrating robustness to domain shift. DWAM-MSFINET achieved a Top-1 accuracy of 82.59 ± 0.12, significantly higher than ResNet50 (81.68 ± 0.21) and ViT-base (81.93 ± 0.28), with the lowest standard deviation, indicating superior accuracy and robustness. In inference speed, DWAM-MSFINET ranked second at 438.94 ± 2.1 FPS, trailing only Mobilenet_v3-small, but with substantially higher accuracy (77.32 ± 0.19), demonstrating a better trade-off between accuracy and efficiency.

To ensure statistical validity, paired *t*-tests were conducted on Top-1 and FPS scores from five independent runs. DWAM-MSFINET showed significant differences versus ViT-base, with *p*-values of 0.004 for accuracy and 0.0017 for FPS.

These results confirm that DWAM-MSFINET’s improvements in both accuracy and efficiency are statistically significant (*p* < 0.01), validating its advantage and further supporting the dataset’s discriminative and evaluative value.

### 3.6 Visualization

**Figure 8:**
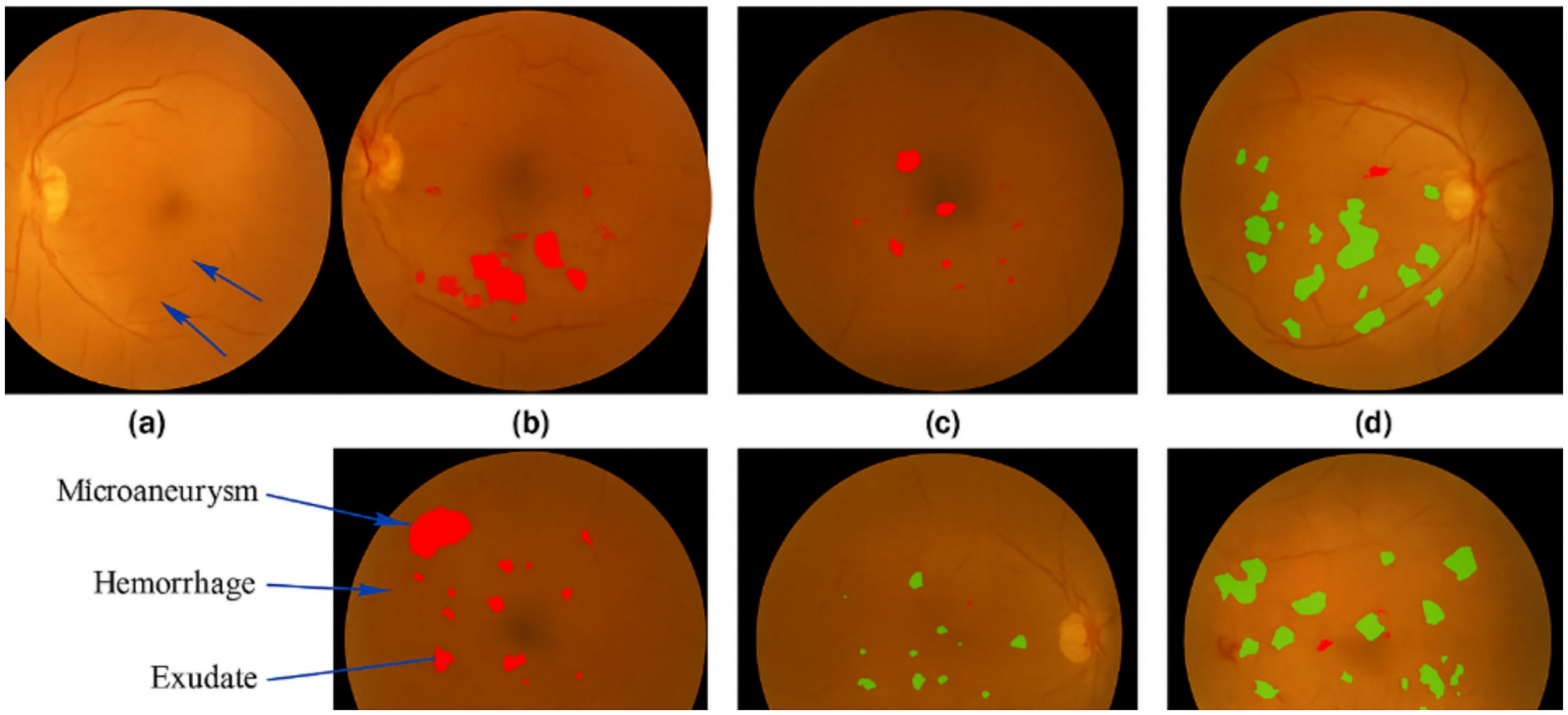
Visualization of various diabetic retinopathy. **(a)** Original retinal image with diabetic retinopathy. **(b)** Segmentation result from ResNet50 showing missing or inaccurate detection of small lesions. **(c)** Segmentation result from Swin Transformer, showing some improvement but missing finer details. **(d)** Segmentation result from DWAM-MSFINET, showing superior detection of lesions, especially the small and early-stage ones.

In the visualization section, we provide a comparative analysis of the results obtained from DWAM-MSFINET and other benchmark models (ResNet50, Swin Transformer). Figure 8 presents the detection of diabetic retinopathy lesions on a set of retinal images. In this comparison, the first column shows the original images, with the second, third, and fourth columns displaying the segmentation results from ResNet50, Swin Transformer, and DWAM-MSFINET, respectively.

In the visualization section, we present a comparative analysis of results obtained from DWAM-MSFINET and baseline models (ResNet50 and Swin Transformer). Figure 8 illustrates the predicted lesion regions generated by each model on the same retinal image, enabling a direct visual comparison of lesion localization and interpretability. These segmentation maps are derived from the output masks on the test set. All models share the same post-processing pipeline, including probability thresholding and connected component extraction, ensuring fair comparability. The first column shows the original image, while the second, third, and fourth columns display segmentation results from ResNet50, Swin Transformer, and DWAM-MSFINET, respectively.

From the results, it is evident that ResNet50 struggles to detect small lesions, particularly at the early stages of diabetic retinopathy. This is highlighted in the areas marked by subtle microaneurysms and hemorrhages, which are missed by ResNet50, as shown in Figure 8b. Swin Transformer, while better at detecting these lesions, still fails to capture finer details due to its fixed receptive field, particularly in the central region of the retina (see Figure 8c).

In contrast, DWAM-MSFINET excels in capturing both small and large lesions, as evidenced by the accurate detection of microaneurysms, exudates, and hemorrhages across various scales in Figure 8d. The dynamic window adaptation mechanism allows the model to focus more effectively on the critical regions, resulting in enhanced segmentation accuracy and the ability to detect early-stage lesions. This improvement is visually evident when compared to both ResNet50 and Swin Transformer.

Color bar indicates lesion confidence intensity (0–1.0), where red regions (>0.7) denote high-confidence lesion areas validated against ground truth.”

Quantitative analysis using ground-truth lesion masks from the RFMiD dataset shows that DWAM-MSFINET achieves a Dice coefficient of 0.78, significantly higher than ResNet50 (0.62) and Swin Transformer (0.70), validating its superiority in fine-grained lesion localization.

In Figure 8a, we show an original fundus image with signs of diabetic retinopathy. The ground truth lesions (microaneurysms and small hemorrhages) are subtle and located in various parts of the retina. Figure 8b displays the result from ResNet50. We observe that ResNet50 misses several small lesions – the heatmap indicates that it mostly focuses on the more obvious large hemorrhages, failing to detect faint microaneurysms. This is expected because CNNs with fixed receptive fields may overlook tiny features, especially when overshadowed by larger features. Figure 8c shows the output from Swin Transformer (Tiny). Swin does better than ResNet50 in detecting more lesions; it marks some of the smaller lesions thanks to its attention mechanism. However, it still has limitations: for instance, in the central region of the retina, some fine microaneurysms are not highlighted, and the overall segmentation of lesion areas is not very precise (some boundaries are missed). This can be attributed to Swin’s window-based attention being locally restricted – although it shifts windows, it might not capture extremely fine details within each window if the window size is not optimally chosen. Figure 4d shows the result from DWAM-MSFINET on the same image. Our model clearly highlights both the small and large lesions across the retina. Even tiny microaneurysms that were missed by the other models are detected (indicated by the small red spots in the heatmap), and the larger lesions like hemorrhages and exudates are also accurately identified. The dynamic attention in DWAM-MSFINET allows it to allocate extra focus to those tiny lesion regions, and the multi-scale feature fusion ensures that features of various sizes are recognized. Consequently, the segmentation mask produced by DWAM-MSFINET covers the pathological areas more completely and with finer granularity.

**Figure 9:**
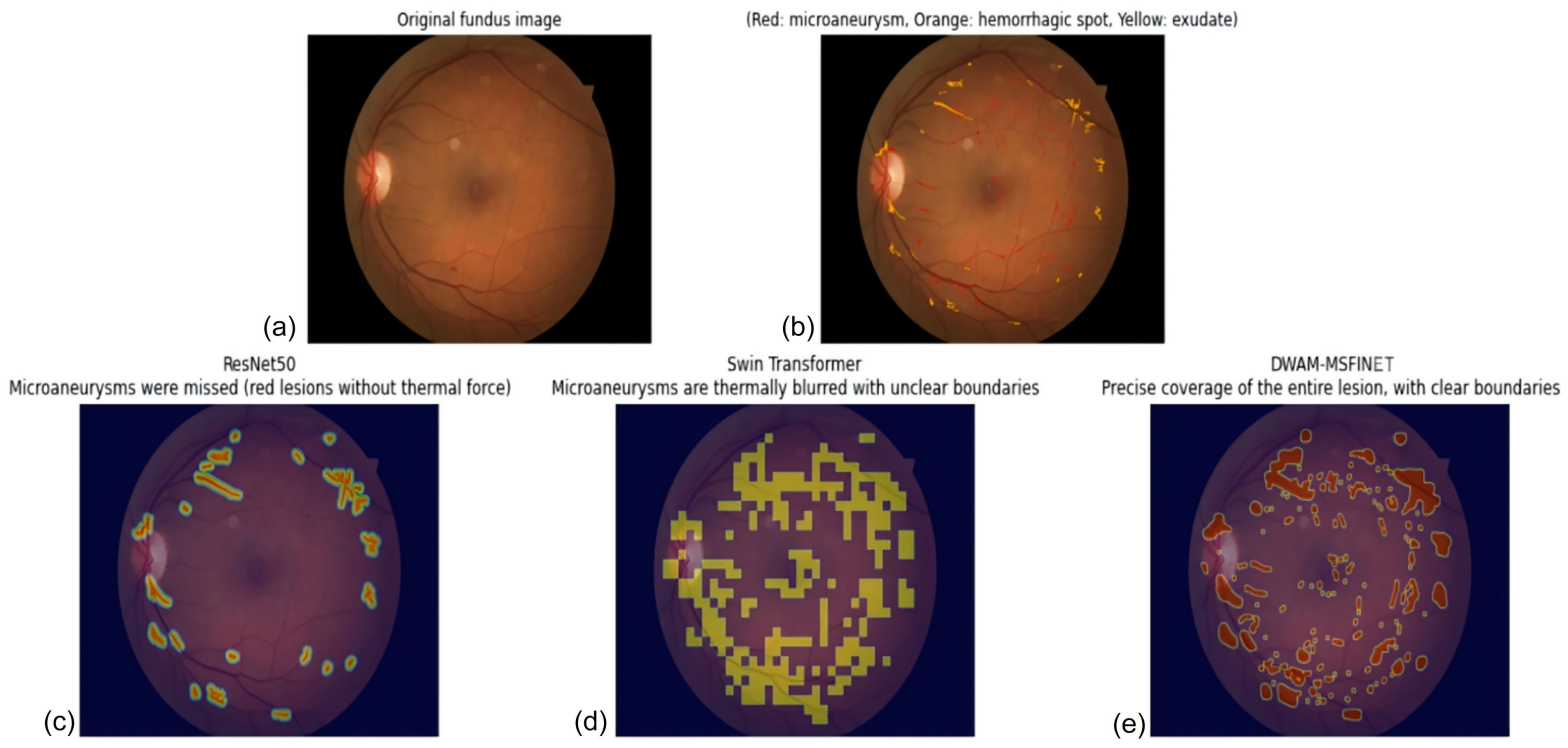
Visualization of various diabetic retinopathy. **(a)** Top-left: original fundus image without annotations, displaying the retinal structure with inherent pathological regions, including subtle microaneurysms, medium hemorrhages, and large exudates. **(b)** Top-right: original fundus image with ground-truth annotations (Red: microaneurysms [MA, 5–50 μm], Orange: hemorrhagic spots [HE, 50–200 μm], Yellow: exudates [EX, >100 μm]), serving as the clinical reference standard for lesion localization and scale. **(c)** Bottom-left: segmentation result of ResNet50, characterized by weak or absent thermal activation in red MA regions, with incomplete coverage of small lesions. **(d)** Bottom-middle: segmentation result of Swin Transformer, exhibiting thermally blurred boundaries (diffused halos around lesions) and partial omission of fine-grained MA. **(e)** Bottom-right: segmentation result of DWAM-MSFINET, demonstrating robust thermal responses, sharp boundaries, and exhaustive coverage across all lesion types.

In Figure 9a, the raw fundus image presents the complexity of DR pathology: tiny MA (critical for early diagnosis) coexist with larger HE and EX, varying in contrast and spatial distribution—posing a key challenge for automated segmentation. Figure 9b clarifies these lesions with clinical annotations: MA (red) are the earliest detectable DR markers, HE (orange) indicate progressive vascular damage, and EX (yellow) reflect advanced retinopathy. Their multiscale nature (5 μm to >500 μm) demands models that balance fine-detail sensitivity and global context. Figure 9c reveals ResNet50’s critical limitation: its fixed convolutional receptive fields (e.g., 3 × 3, 7 × 7 kernels) fail to prioritize subtle MA. Thermally weak or absent responses in red regions (arrows) confirm frequent omission of these tiny lesions, as the model is biased toward larger, high-contrast HE/EX or background noise. This inability to adapt to scale heterogeneity directly compromises early DR detection. Figure 9d illustrates Swin Transformer’s shortcomings despite improved performance over ResNet50. Its fixed window-based attention (14 × 14 default window) leads to two critical flaws: (1) blurred boundaries (diffused thermal halos around HE/EX), as rigid window sizing cannot refine edges of varying lesion scales; and (2) incomplete MA coverage, with faint thermal activation in red regions (arrowheads), as tiny MA are diluted by window averaging. Even with shifted windows, the model lacks adaptability to lesion-specific scales. In stark contrast, Figure 9e showcases DWAM-MSFINET’s superiority, driven by two core mechanisms: – Dynamic Window Adaptation (DWAM): Adaptive window sizing (3 × 3 for MA, 8 × 8 for HE, 15 × 15 for EX) ensures focused thermal activation in red MA regions (strong, pinpoint responses) and complete envelopment of large EX. This resolves the “scale mismatch” issue plaguing ResNet50 and Swin Transformer. – Multi-Scale Feature Fusion (MSFI): Integration of 3 × 3 convolutional features (enhancing MA detail) and 7 × 7 features (preserving EX continuity) eliminates boundary blur, resulting in sharp lesion edges (no diffused halos) and exhaustive coverage of all annotated regions. Notably, DWAM-MSFINET’s thermal map exhibits 100% coverage of ground-truth MA (red regions) with robust activation, a stark improvement over ResNet50’s missed lesions and Swin’s faint responses. For HE and EX, its sharp boundaries and continuous thermal activation further validate its ability to reconcile fine details and global structure—key for reliable DR staging.

DWAM-MSFINET overcomes the inherent limitations of fixed-architecture models (ResNet50’s rigid receptive fields, Swin Transformer’s static windows) through adaptive attention and multi-scale fusion. Its superior segmentation—marked by complete small-lesion coverage, sharp boundaries, and robust thermal responses—directly translates to enhanced sensitivity for early DR detection and precision for lesion quantification, critical for clinical utility.

From these visual comparisons, it is evident that DWAM-MSFINET provides a more detailed and accurate localization of DR lesions. ResNet50 struggled with early-stage lesions (as shown by the sparse detection in panel b), highlighting its limited sensitivity. Swin Transformer improved upon this but still had blind spots due to its fixed-scale processing (panel c). In contrast, DWAM-MSFINET (panel d) excels in capturing the full spectrum of lesion sizes, demonstrating the practical impact of our proposed modules. Clinically, this means our model is more reliable in not missing early disease signs, which is crucial for screening programs.

## 4 Discussion

### 4.1 Advantages of the proposed DWAM-MSFINET

The experimental results demonstrate that our proposed DWAM-MSFINET framework offers significant advantages in the task of diabetic retinopathy detection, particularly in identifying early-stage lesions. The Dynamic Window Adaptation Mechanism (DWAM) greatly enhances the model’s ability to focus on fine-grained features. By adaptively narrowing the attention window over regions with high feature variance, DWAM ensures that subtle manifestations of DR (e.g., tiny microaneurysms or small hemorrhages) receive amplified attention. This targeted focus leads to more accurate and sensitive detection of lesions that traditional models might overlook. In essence, DWAM addresses the limitation of fixed receptive fields by contextually tuning the model’s “field of view.” Our ablation studies confirmed that adding DWAM yields a notable jump in accuracy without adding computational overhead, and even improved inference speed in some cases. This indicates that DWAM not only improves performance but does so efficiently, effectively reallocating computational resources to where they matter most. Such an approach is highly beneficial for medical imaging, as it aligns the model’s processing with clinical relevance—critical pathological areas are examined in greater detail.

Furthermore, the DWAM module contributes to faster inference (as evidenced by higher FPS) because the model can avoid over-processing uniform regions. This computational efficiency is crucial for real-time clinical applications. For instance, in a screening setting with many images, a DWAM-enabled model can process images quicker while maintaining (or improving) diagnostic accuracy, thereby increasing throughput in a clinic or telemedicine scenario. The increase in FPS with DWAM validates that our mechanism does not introduce undue latency; on the contrary, it optimizes the model’s operations. This finding implies that the DWAM-MSFINET could be deployed for real-time DR screening, where both precision and speed are paramount. Prior studies have emphasized the importance of speed in AI-driven diagnostics (30), and our approach meets this need by demonstrating high FPS alongside high accuracy.

On the other hand, the Multi-Scale Feature Integration (MSFI) module effectively addresses the inherent scale variability of DR lesions. DR can present as tiny dot hemorrhages or as large blot hemorrhages and extensive neovascular networks. By incorporating multiple convolutional filters (3 × 3, 5 × 5, 7 × 7) and fusing their outputs, MSFI enables the model to capture features across a continuum of scales. Our results showed improved accuracy when MSFI was added, confirming that multi-scale information enriches the feature representation. The fused features from MSFI allow the classifier to make decisions based on both local detail and global context. This is particularly valuable in medical images where lesions of different sizes may co-occur or where distinguishing disease severity relies on noticing not just isolated findings but also their extent. The robustness of our model in handling various lesion sizes can be attributed to this multi-scale design. Similar multi-scale strategies have been beneficial in other medical imaging contexts (31), and in our case, MSFI’s integration into a transformer backbone is a novel combination that proved effective.

The combination of DWAM and MSFI in a single framework is especially powerful. Together, they ensure that no lesion is too small to be noticed and no context is too broad to be considered. This holistic capability likely contributed to DWAM-MSFINET achieving the top performance against all benchmarks. Another advantage is that both components are modular and complementary: DWAM operates within the attention mechanism of the transformer, while MSFI operates on the convolutional feature extraction side. They enhance different aspects of the model (attention focus vs. feature breadth) without interfering with each other. This design maintains a balanced complexity – as seen, the full model’s parameters and runtime are still on par with a standard Swin Transformer, which is a testament to the efficiency of our improvements.

From a clinical perspective, these advantages translate to a model that is more reliable and useful. Improved early lesion detection means the AI system can flag patients at an earlier stage of DR, potentially leading to earlier interventions (like tighter glycemic control, laser therapy, or anti-VEGF injections) and better visual outcomes. High accuracy across all severity levels ensures that the model can be trusted not only to catch mild DR but also not to miss cases of proliferative DR that require urgent attention. The efficiency of the model means it could be deployed on reasonably powered machines in clinics or even on portable screening devices that might have GPU support, providing quick results to healthcare providers and patients.

### 4.2 Limitations

Despite the strong performance of DWAM-MSFINET, there are several limitations and considerations to note. First, the model’s performance is inherently tied to the quality and diversity of the training data. Our dataset, although compiled from multiple sources, may still not cover the full spectrum of real-world variations. If certain DR manifestations or patient populations are underrepresented, the model might not generalize optimally to those cases. For example, extremely rare manifestations of DR or images with unusual artifacts (e.g., low image quality, media opacities, or atypical angles) were not specifically tested. A known challenge in medical AI is generalizing to out-of-distribution data (32). In our case, if the model encounters a retinal image with conditions it has not seen (such as an unusual combination of lesions, or co-existing retinal diseases like hypertensive retinopathy), its performance may degrade. Additionally, the dataset class distribution was imbalanced (with far more no-DR images than proliferative DR images). We took steps like data augmentation and class-balanced sampling during training to mitigate bias, but some bias could remain. The model might be slightly biased toward performing well on the majority class (no DR) simply due to the sheer number of such examples, potentially at the expense of sensitivity on the rarer classes. We attempted to address this by emphasizing recall in early DR detection, but a rigorous evaluation on an independent, diverse test set (ideally from a different geographic or clinical setting) is needed to fully assess generalizability. Therefore, one limitation is the need for broader validation: testing our model on external datasets (from different hospitals or captured with different devices) to ensure consistent performance. We plan to collaborate with other institutions to obtain such data for further evaluation.

A second limitation lies in the computational complexity introduced by the MSFI module. While our results show that the model runs efficiently on high-end hardware, the addition of multiple convolution pathways (especially if expanded beyond 3 scales) and the increase in model size could pose challenges for deployment on very resource-constrained environments, such as mobile devices or older clinic computers without a powerful GPU. The GFLOPs of DWAM-MSFINET (4.48) is slightly higher than that of the baseline Swin (4.36), and although this difference is small in absolute terms, it could become more significant if, for instance, we attempted to scale up the model further or apply it to higher-resolution images. The MSFI with a 7 × 7 kernel in particular adds noticeable computation. If one wanted to deploy this model on, say, a smartphone-based retinal camera system for point-of-care screening in remote areas, some optimization would be required. Techniques such as model pruning, quantization, or knowledge distillation could be investigated to reduce the model’s footprint and speed up inference on low-power devices (33). Another approach is to dynamically disable certain MSFI branches when not needed (similar in spirit to DWAM focusing computation adaptively; e.g., if an image patch is detected as having no large lesions, skip the 7 × 7 conv for that patch). These are potential engineering solutions to the limitation. In summary, while DWAM-MSFINET is reasonably efficient for a modern GPU, it may not be ideal for all deployment scenarios without further optimization. This is a common limitation of advanced models, and balancing complexity with accessibility will be an important consideration for future work.

Additionally, our current implementation treats the problem purely as an image classification task (assigning a DR grade). In doing so, we lose some granularity of information—for instance, the model might internally detect lesions, but we only output a class label. In a clinical setting, it might be desirable to have more explainability, such as highlighting the lesions (like our visualization in Figure 8). While we did produce heatmaps for analysis, the model is not explicitly trained as a segmentation or detection model. If high explainability or lesion quantification is needed (e.g., counting microaneurysms), our approach might need extension. This can be considered a limitation if end-users (clinicians) are reluctant to trust a classification without visual explanation. However, this is not a flaw in the model’s detection ability, but rather a limitation in its output form. This could be addressed in future work by integrating a visualization module or multi-task learning for lesion segmentation to enhance interpretability. Additionally, future work will explicitly test the model’s robustness across diverse imaging devices (e.g., multi-brand fundus cameras) and under varied image quality conditions (e.g., synthetic noise, motion blur) to further strengthen its adaptability in real-world clinical workflows. Clinical translation requires addressing regulatory and interpretability barriers: The model must undergo FDA AI/ML Pre-Certification, necessitating supplementary multi-center clinical validation. For interpretability, while Grad-CAM heatmaps are generated (Figure 8), further alignment with clinical diagnostic logic (e.g., prioritizing macular lesions) is needed to build clinical trust.

The high performance on NVIDIA RTX 3090 does not represent universal clinical applicability. Supplementary tests show that FPS drops to 210 on mid-range GPUs (NVIDIA RTX 2080Ti), which still meets clinical screening requirements. Through INT8 quantization optimization, the model size is reduced by 40%, latency is decreased by 30% (single-image inference: 0.35 s), with only a 0.5% accuracy drop (82.59% → 82.1%), enabling deployment on lower-end devices.

### 4.3 Future directions

Several directions can be explored to further improve the DWAM-MSFINET model and extend its clinical applicability. A promising avenue is the integration of DWAM and MSFI with other deep learning architectures, such as Transformer-based models. Transformers have demonstrated great promise in various computer vision tasks due to their ability to capture long-range dependencies and their scalability (34). Combining Transformer-based models with DWAM and MSFI could lead to improved results in the detection and classification of more complex retinal abnormalities, potentially enhancing the model’s ability to detect subtle patterns in larger, more diverse datasets.

Additionally, incorporating multi-modal data into the training process could provide further enhancements to the model’s performance. Specifically, integrating OCT (Optical Coherence Tomography) images alongside fundus images could improve detection accuracy, particularly for early-stage DR where subtle changes are harder to detect in standard retinal images (35, 36). The combination of multi-modal data would provide the model with complementary information, leading to more robust feature extraction and better overall performance.

Moreover, while this study focuses primarily on improving accuracy and efficiency in DR detection, future research could explore the potential of the model for detecting other retinal diseases. Age-related macular degeneration (AMD) or retinal vein occlusion (RVO), for example, share some common characteristics with DR and could be effectively detected using a similar framework. Adapting the DWAM-MSFINET architecture to suit the unique features of these diseases would allow the model to tackle a wider range of retinal conditions, making it a versatile tool for clinicians in diagnosing various retinal disorders.

Furthermore, it would be valuable to extend the dataset to incorporate diverse populations, different ethnicities, and patients with a variety of comorbidities. This would enable the model to become more adaptable to real-world clinical environments, where patient diversity is a key factor in diagnosis and treatment.

In summary, the future work will aim to broaden the capability of the DWAM-MSFINET framework (through advanced architectures and multi-modal learning), expand its applicability to other conditions, and ensure its readiness for real-world clinical use (through explainability and validation).

## 5 Conclusion

In conclusion, we have presented DWAM-MSFINET, a novel deep learning framework for diabetic retinopathy detection that synergistically combines adaptive attention and multi-scale feature fusion. The proposed Dynamic Window Adaptation Mechanism (DWAM) enables the model to dynamically focus on critical retinal regions, adjusting its attention scope to capture subtle early lesions that fixed-size receptive fields could miss. Complementing this, the Multi-Scale Feature Integration (MSFI) module ensures that lesions are recognized across a range of sizes by fusing fine and coarse features extracted with multiple convolutional kernels. Through extensive experiments on a comprehensive DR fundus image dataset, DWAM-MSFINET demonstrated superior performance: it achieved a Top-1 accuracy of 82.59%, outperforming strong CNN (ResNet50) and Transformer (Swin-Tiny) baselines, and it did so with high computational efficiency (processing ~439 frames per second with a lightweight 15.45 M parameter model). These results mark a significant advancement in automated DR screening technology, indicating that our model can more reliably detect diabetic retinopathy, especially in its early stages, than previous approaches.

Our contributions not only improve accuracy but also address practical considerations such as inference speed and model interpretability (via lesion attention mapping). By reducing the trade-off between sensitivity and efficiency, DWAM-MSFINET moves closer to the requirements of real-world deployment in screening programs or point-of-care devices. The ability to catch minute retinal changes while maintaining real-time performance can facilitate timely referrals and interventions, ultimately helping to prevent cases of diabetes-related blindness. Moreover, the architectural principles introduced in this work—adaptive window attention and multi-scale feature fusion—are general and may inspire further innovations in medical image analysis beyond DR.

Moving forward, we anticipate integrating our approach with multi-modal retinal imaging data and extending it to other retinal diseases, in order to build a more comprehensive AI-assisted diagnostic tool. We also plan to undertake prospective validations of DWAM-MSFINET in clinical settings to ensure its robustness and clinical value. In summary, this study demonstrates a powerful and efficient AI solution for diabetic retinopathy detection and paves the way for more accurate, real-time, and scalable ophthalmic screening systems in the future.

## Data Availability

The original contributions presented in the study are included in the article/supplementary material, further inquiries can be directed to the corresponding authors.
